# Poor body condition is associated with lower hippocampal plasticity and higher gut methanogen abundance in adult laying hens from two housing systems

**DOI:** 10.1038/s41598-022-18504-1

**Published:** 2022-09-15

**Authors:** E. A. Armstrong, P. Richards-Rios, L. Addison, V. Sandilands, J. H. Guy, P. Wigley, T. Boswell, T. V. Smulders

**Affiliations:** 1 Centre for Behaviour and Evolution, Newcastle University, Newcastle upon Tyne, UK; 2 Biosciences Institute, Newcastle University, Newcastle upon Tyne, UK; 3 Institute of Infection, Veterinary and Ecological Sciences, University of Liverpool, Liverpool, UK; 4 School of Natural and Environmental Sciences, Newcastle University, Newcastle upon Tyne, UK; 5 Monogastric Science Research Centre, SRUC, Edinburgh, UK

**Keywords:** Neuroscience, Neurogenesis, Microbiome

## Abstract

It is still unclear which commercial housing system provides the best quality of life for laying hens. In addition, there are large individual differences in stress levels within a system. Hippocampal neurogenesis or plasticity may provide an integrated biomarker of the stressors experienced by an individual. We selected 12 adult hens each with good and poor body condition (based on body size, degree of feather cover and redness of the comb) from a multi-tier free range system containing H&N strain hens, and from an enriched cage system containing Hy-Line hens (n = 48 total). Immature neurons expressing doublecortin (DCX) were quantified in the hippocampus, contents of the caecal microbiome were sequenced, and expression of inflammatory cytokines was measured in the spleen. DCX^+^ cell densities did not differ between the housing systems. In both systems, poor condition hens had lower DCX^+^ cell densities, exhibited elevated splenic expression of interleukin-6 (*IL6*) mRNA, and had a higher relative caecal abundance of methanogenic archea *Methanomethylophilaceae*. The findings suggest poor body condition is an indicator that individual hens have experienced a comparatively greater degree of cumulative chronic stress, and that a survey of the proportion of hens with poor body conditions might be one way to evaluate the impact of housing systems on hen welfare.

## Introduction

While conventional cages are no longer permitted in the European Union (EU Council Directive 1999/74/EC), current types of commercial housing for laying hens still differ in notable ways, and comparisons of relative stressful experience for birds within them have yielded mixed results. Laying hens are housed either in enriched cages, or in non-cage systems, which may provide outdoor access^1^. Non-cage systems comprise a litter floor and either single-tier structures (containing nest boxes, feeders, drinkers), or multi-tier structures with resources spread across several levels. The systems differ in parameters including average group sizes, environmental complexity, exposure to parasites and disease, challenges to bone health/integrity, opportunities for a naturalistic behavioural repertoire (e.g. foraging, comfort & vertical-plane behaviours) and incidences of deleterious behaviours (e.g. cannibalism, piling and smothering)^2^. Average mortality is higher in non-cage (i.e. barn, free-range and organic) systems than in cages^3,4^. Behaviourally, hens from conventional and enriched cages appear to be more acutely fearful than non-cage birds^1,5^, though hens kept in floor pens, single-bird and 5-bird cages displayed comparable durations of tonic immobility, shorter than for 17-bird cages^6^, implying lower anxiety levels for the smaller groups. Physiologically, observed differences in basal levels of plasma corticosterone (Cort) between cage and non-cage systems are not consistent in their direction between studies^7–12^. Heterophil:lymphocyte (H:L) white blood cell ratios are lower in hens provided experimentally with range access^13^, suggesting an anti-stress effect, but observational studies have not found a difference in H:L ratios between enriched cages and free-range systems^14,15^. The majority of assessments have focused upon measures of acute stress. Application of an integrated biomarker to compare the collective longer-term stressful experience associated with each type of housing would provide further insight into the welfare qualities of the different housing systems.

Consumer concern often focuses on the consequences of housing structure (particularly use of cages and range access) for group welfare^16^, but differences in the experiences of individual hens within systems may also have significant implications. Within genetic strains, hens differ in terms of intrinsic traits (e.g. health^4^, innate immunity^17^ and susceptibility to disease^18^, responsiveness to stress^19^, preferences^20^, personality and cognition^21^) and their non-shared experiences within production systems (pathogen exposure^2^, injuries^22,23^, social interactions^24^, properties of the proximal environment/cage location^25^, etc.). Whether such intra-flock variation influences the cumulative stressful experience of hens within specific systems, and contributes to whether individuals have lives worth living, warrants further exploration. Observable phenotypic characteristics are generally easier to assess than behaviour or physiology, particularly in commercial settings. Studies suggest that physical and psychological stress are associated with hens exhibiting lower body weights^26–30^ and poorer feathering^31–37^, while birds that are fearful^38^ and subordinate^39,40^ tend to have paler combs. However, it is not clear whether the overall cumulative welfare of these poor condition hens is worse than that of flock-mates in good conditions at the end of lay. If physical body condition can be associated with a validated marker of long-term stress, it may be employed as an easily assessable proxy for welfare status. How individual factors compare to shared effects of the housing environment in magnitude is also not known, and production systems may differ in the extent to which they favour certain characteristics over others. For example, individual differences in immunity may be more consequential where pathogen exposure is greater, and the importance of socially relevant traits may differ with group size.

Studies in rats and mice suggest that adult hippocampal neurogenesis provides an integrated measure of positive and negative experiences: levels are highest following multiple positive experiences^41^, intermediate (and comparable to control conditions) following combined positive and negative experiences^42–47^, and lowest following multiple negative experiences^48^. In rodents, new hippocampal neurons are often identified by their expression of plasticity marker doublecortin (DCX), which labels a mixed population of immature neurons. In the rodent hippocampus, staining for DCX has been reported to produce similar estimates of cell numbers to BrdU labelling^49^, including with respect to changes in neurogenesis rates that occur in response to behavioural experiences^50^. DCX has also been used in birds, most notably in the song system, where it labels neurons ranging from 3 to 30 days old^51,52^. However, in birds, there is still a debate about whether DCX only marks new neurons, or more generally neurons with high plasticity^53–55^. We therefore err on the side of caution and refer to DCX staining as representing neural plasticity, which includes newly-generated neurons and potentially also other neurons expressing morphological plasticity. Recently, DCX^+^ neurons in the hippocampus have been employed as a biomarker of chronic stressful experience in chickens, being quantitatively suppressed by experimental unpredictable stress^56^, severe keel bone fractures^57^ and long-term food restriction^58^. In terms of negative experience, inflammation is a further physiological sign of chronic stress and depressive-like mood^59^, and challenges to health and immunity^60^, across species. In chickens, quantitative PCR has been used to measure the effect of Cort administration on the expression of cytokines, chemokines, and their receptors, providing a marker of the effects of stress on the immune system^61^. Composition of the intestinal microbiome (the population of microbes that make up an ecosystem within the gut) is also responsive to physical and psychological stress^62,63^, and has been linked to health and welfare in chickens, in particular through microbial dysbiosis^64,65^. Interactions between the brain, gut and microbiome influence laying hen behaviour^66^, as chicken lines displaying high and low levels of feather pecking are characterised by divergent microbiota^67,68^. Research on the effect of housing system on the microbiome in hens is currently limited, though one descriptive study indicated differences between caged and free-range systems^69^.

By measuring biomarkers of longer-term stress in hens with good and poor physical body conditions (body size, feather cover & comb colour) sampled from both a commercial enriched cage system and a multi-tier aviary with outdoor range, we sought to determine whether: (1) one housing system offers superior welfare for birds at both extremes of the body condition spectrum, (2) there is overlap in experience between systems relating to physical individual differences, or (3) birds in similar conditions have comparable experiences in different housing environments. In terms of outcome measures, adult hippocampal plasticity was assessed by quantifying densities of immature neurons expressing differentiation-marker doublecortin (DCX) in serial hippocampal sections, contents of the caecal microbiome were sequenced to determine relative abundance of different bacterial clades, and inflammatory gene expression was measured in the spleen.

## Methods

### Ethical statement

The study was approved by the Animal Welfare and Ethical Review Body at Newcastle University (Project ID #702), and all methods complied with UK regulations regarding the treatment of animals. Birds were housed and managed according to RSPCA-Assured standards and DEFRA guidelines on farm, and the Home Office Code of Practice while at the University. A Home Office Schedule 1 method of euthanasia was used. Reporting of the study follows the recommendations in the ARRIVE guidelines.

### Adult hens

From one day old, H&N and Hy-Line Brown pullets (*Gallus gallus domesticus*) were reared in two floor-based systems with litter at commercial farms in the Midlands, UK. Both sites were operated by the same pullet rearing company, Country Fresh Pullets, according to RSPCA-Assured standards, and arrived at an egg production farm, operated by a different company, in northern England in October 2017. The H&N pullets were 16 weeks of age when introduced to a multi-tier free range adult housing system, whilst the Hy-Line birds were 17 weeks of age upon introduction to an enriched cage system on the adjacent site. At the time of sampling in October 2018, both groups had spent almost a year living in their respective systems, and hens were 65 weeks of age in the multi-tier system and 66 weeks of age in the enriched cage system.

The multi-tier housing unit consisted of 16,000 H&N strain birds, divided into four internal colonies of 4000 hens. However, as these birds all shared a range, individuals could move between the colonies during the day by accessing different popholes. Within the shed, the floor was covered with wood shavings litter and there were three additional tiers, the top of which was located 2.4 m above the floor. The system provided round metal perches and nest boxes shaded by orange plastic dividers. Internal stocking density was 9 birds/m^2^ of usable area, where usable area is made up of the ground surface of the building accessible to the hens and additional raised areas and platforms at least 30 cm wide. Water was provided through nipple-drinkers, with one nipple for every 10 birds. Layer feed was circulated through a conveyor belt system, with a frequency of eight times per day. The average temperature inside the shed was 19–22 °C. Birds received 15.75 h of artificial light per day, from 06:45 to 22:30. Popholes opened at 09:00 daily and were closed 30 min after twilight ended. The grassy range had a dimension of 8.1 hectares and contained several two-tiered, covered wooden shelters, with ramps to the upper tier. No cover was provided by vegetation.

The enriched cage housing unit contained 33,120 Hy-Line strain birds, with a stocking density of 15 birds/m^2^. Each cage was 300 (l) × 150 (w) × 55 (h) cm in dimension and held 50 birds. Cages were arranged into four banks of three vertical tiers with 22 cages per tier, repeated over two floors. Enrichment provided in each cage consisted of perches, a nest box, a scratch mat and a grit auger to drop feed onto the scratch mat. The average temperature in the unit was 19–23 °C. Birds received 15.5 h of artificial light per day, from 01:45 to 17:15. Water was provided through nipple-drinkers, with one nipple for every 10 birds. Layer feed was circulated six times a day through a conveyor belt system. Birds in both systems experienced an identical programme of vaccinations. Hens in the multi-tier system were given a wormer at four intervals during lay. Due to poor laying performance, enriched cage birds alone were given a course of antibiotics at 39 weeks of age (Denegard; for the treatment and prevention of chronic respiratory disease and air sacculitis caused by *Mycoplasma gallisepticum* and *Mycoplasma synoviae*). Feed intake was typically 20 g per bird higher per day in multi-tier system than in the enriched cages. At 65 weeks of age (for comparability), average bird mass was 1544 g in the multi-tier aviary compared to 1967 g in the enriched cage system. Production rates were 90.3% in the multi-tier and 81.6% in the enriched cage system, and cumulative mortality had reached 2.50 and 2.74% respectively.

Whilst the multi-tier free-range and enriched cage housing systems were operated by separate personnel, they were overseen by the same Production Manager, whose role was to promote the efficient and sustainable production of eggs for retail sale.

### Sampling

**Figure 1:**
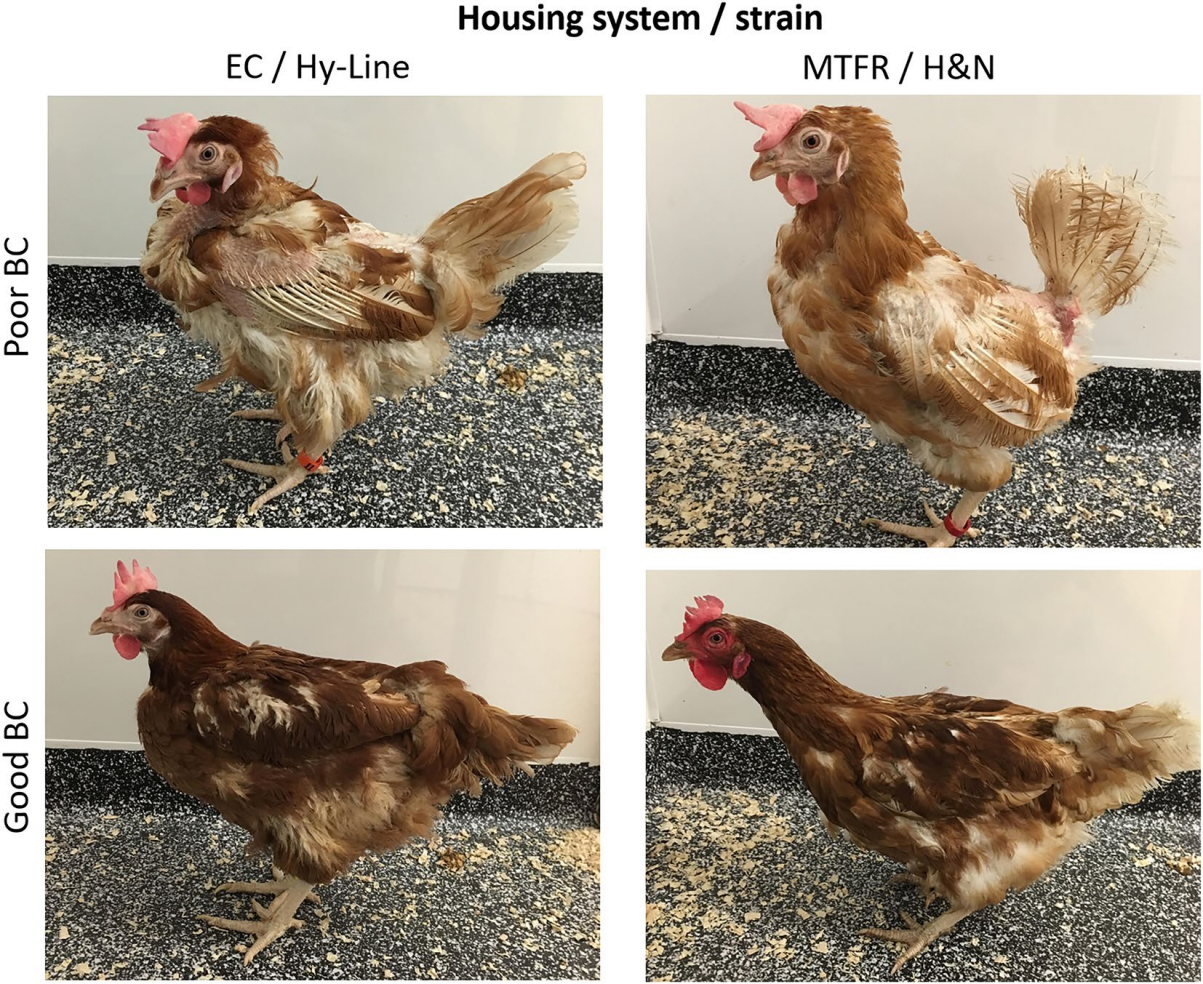
Representative images of hens of both strains (Hy-Line and H&N) with good and poor body conditions (BCs) sampled from the two housing systems: enriched cages (EC) and multi-tier aviary with free range (MTFR).

Within each housing system, birds with good and poor external indicators of physical body condition were selected. Hens were chosen by the farm’s Production Manager, who was familiar with the conditions of the birds. The a priori criteria employed were: (1) body size, (2) feather coverage, and (3) redness of the comb, wherein a high level of each factor represented good physical condition. Because keel bone damage was previously found to be associated with reduced adult hippocampal plasticity^57^, candidate hens were palpated by the same researcher (EAA), trained to an agreed standard^70^, and those displaying signs of damage were rejected. A total of 12 hens with good body condition and 12 hens with poor body condition were sampled from both the multi-tier and enriched cage system, equating to a total sample size of 48 birds. This group size has proved sufficient to detect experience-based differences in our primary outcome measure (DCX^+^ cell densities) in previous studies^56,57^. While the intention had been to sample hens of the same strain from the two housing systems, this was not logistically possible. Sampling occurred over four successive days, upon each of which 12 birds (3 × multi-tier/H&N: good condition, 3 × multi-tier/H&N: poor condition, 3 × enriched cage/Hy-Line: good condition, 3 × enriched cage/Hy-Line: poor condition) were selected from the farm and transported in carry boxes to Newcastle University for processing on the same day. In order to capture extremes of experience in the multi-tier system, good condition birds were selected directly from the range, while poor condition birds were selected from the top inside tier. This meant that the sampled good condition birds ventured outside at least some of the time, whilst the sampled poor condition birds may or may not have chosen to range. To allow birds time to go outside, there was a 30-min delay between the opening of pop-holes in the morning and sampling of birds from the range. Hens were sampled from a different internal colony of the shed on each of the four days, along with the area of the range proximal to these colonies. To ensure independence in the enriched cage system, no two birds were sampled from the same cage, but to avoid a potential confound of cage height, a good and poor condition bird were always selected from different cages on the same tier. To collect a sample representative of the whole housing system, birds were sampled from each tier (top, middle and bottom) in both an inside and outside bank on each of the two floors. Representative images of good and poor physical condition hens sampled from each housing system are displayed in Fig. 1.

### Sampling of representative pullets at the rearing stage

As different strains of brown hens were sampled from the two adult housing systems, thereby confounding strain and housing type, a sample of pullets of the same genotypes were also taken directly from the rearing farm, to provide a baseline for possible genetic influences on the other measures taken. This sampling occurred after that of the adult hens, in January 2019. H&N and Hy-Line pullets were housed from one day-old in adjacent barns of a commercial rearing farm in Shropshire, England, operated by Country Fresh Pullets according to RSPCA-Assured standards^71^. The chicks originated from separate (hybrid-specific) hatcheries, each in the west of England. Both housing sheds contained litter in the form of wood shavings (Easichik, UK), which were bedded to a depth of 7.6 cm at the point chicks were introduced. Raised slatted areas were provided for perching, with access assisted by ramps placed every 7.6 m along their length. The H&N shed had a total area of 1187.92 m^2^ (including the floor and raised slatted areas) and contained 17,085 pullets, which equated to a stocking density of 14.4 birds/m^2^. Though similar in design, the Hy-Line shed had a larger total area of 1722.12 m^2^ and housed 25,236 pullets, with a stocking density of 14.7 birds/m^2^. Water was provided via nipple drinkers, with 12.2 and 12.4 birds per drinker in the H&N and Hy-Line sheds respectively. Both sets of birds had access to feed at all times via a chain feeder, but the quantity of feed in the feeder and the number of times it was topped up per day was adjusted throughout rearing, in order to maintain target body mass for each flock. The H&N shed housed 39 birds per metre of chain feeder, compared to 37 birds in the Hy-Line shed. Both strains received 10 h of artificial light per day from six weeks of age onwards, with a light intensity of 10 lx at bird height. Ambient temperatures were 32–33 °C for at the introduction of day-old chicks and were gradually reduced by 0.5 °C per day, before being maintained at 20 °C. Both strains experienced an identical program of vaccinations, administered from day-old to 13 weeks. At the time of sampling, average bird mass was 1203 g for the H&N flock and 1239 g for the Hy-Line flock. Both flocks had a cumulative mortality of 1.9%. Sampling of pullets occurred during a single day, when both strains of bird were 14 weeks and 3 days old. Twelve birds of average size and body condition were selected from each rearing barn by a senior Production Manager (total n = 24) and manually palpated by EAA to determine if keel bone fractures were present. Individuals exhibiting damage were rejected. Animals were placed in carry boxes and transported to Newcastle University, where they were housed in two pens (one HyLine, one H&N) with ad libitum feed and water, prior to tissue collection over the following two days (12 birds/day).

### Tissue collection and processing

Collection of tissue from the adult hens and pullets occurred in two separate phases (in October 2018 and January 2019, respectively). Animals were weighed before being killed with an intravenous injection of pentobarbital (Euthatal, 0.5 ml/hen), according to a schedule that alternated between housing system/strain and body condition for the adult hens (n = 48) and strain for the pullets (n = 24). Blood samples were collected for analysis of DNA methylation (results to be reported elsewhere). The spleen was removed and weighed before a sample was placed on ice in a tube containing 1 ml RNAlater® Stabilization Solution (Thermo Fisher Scientific, Loughborough, UK). The caecum was dissected from the gut and placed into a 15 ml Falcon tube before freezing on dry ice. Simultaneously, the brain was removed from the skull, placed into 0.1 M phosphate-buffered saline (PBS) in a Petri dish and divided along the longitudinal fissure with a scalpel. The forebrain hemisphere collected for immunohistochemical analysis alternated between left and right, in a manner that was balanced within groups of hens of each body condition and from each housing system. This tissue was immersion fixed for 44–48 h in 4% paraformaldehyde in 0.5 M PBS at 4 °C. Samples were then cryoprotected in a solution of 30% sucrose in 0.5 M PBS, before being embedded in OCT (4583, Electron Microscopy Sciences—USA). Coronal Sects. (50 μm) were cut on a cryostat (HM 550, Microm—Germany) and stored in cryoprotectant solution (30% glycerol, 30% ethylene glycol, 0.1 M PBS) at − 20 °C. Serial sections taken at 400 μm intervals were processed for immunohistochemistry.

### Immunohistochemistry and quantification of adult hippocampal plasticity

As previously^56,57^, hippocampal formation (HF) tissue sections were stained using an antibody to doublecortin (DCX), to allow quantification of immature neurons. Free-floating sections from the adult hens were stained over six batches, each of which contained tissue from eight birds (2 × multi-tier/H&N: good condition, 2 × multi-tier/H&N: poor condition, 2 × enriched cage/Hy-Line: good condition, 2 × enriched cage/Hy-Line: poor condition). Sections from the pullets were stained over three batches, each of which contained tissue from eight birds, wherein four were of the H&N strain and four were Hy-Line. Staining was conducted according to the protocol detailed in Armstrong et al.^57^. The primary antibody was rabbit polyclonal to doublecortin (Abcam Cat# ab18723, RRID:AB_732011), incubated at a concentration of 1:1000 for 18 h (4 °C). Secondary antibody incubation utilised biotinylated antirabbit IgG secondary antibody made in goat (Vector Labs, BA-1000), at concentration 1:500 for 120 min (room temperature). 1:250 Horse Radish Streptavidin (Vector Labs, SA-5004) was used for conjugate enzyme incubation, and 3,3′-Diaminobenzidine (DAB) SIGMAFAST tablets were dissolved in pure water (final concentration 0.35 mg/ml) for chromogen incubation.

**Figure 2:**
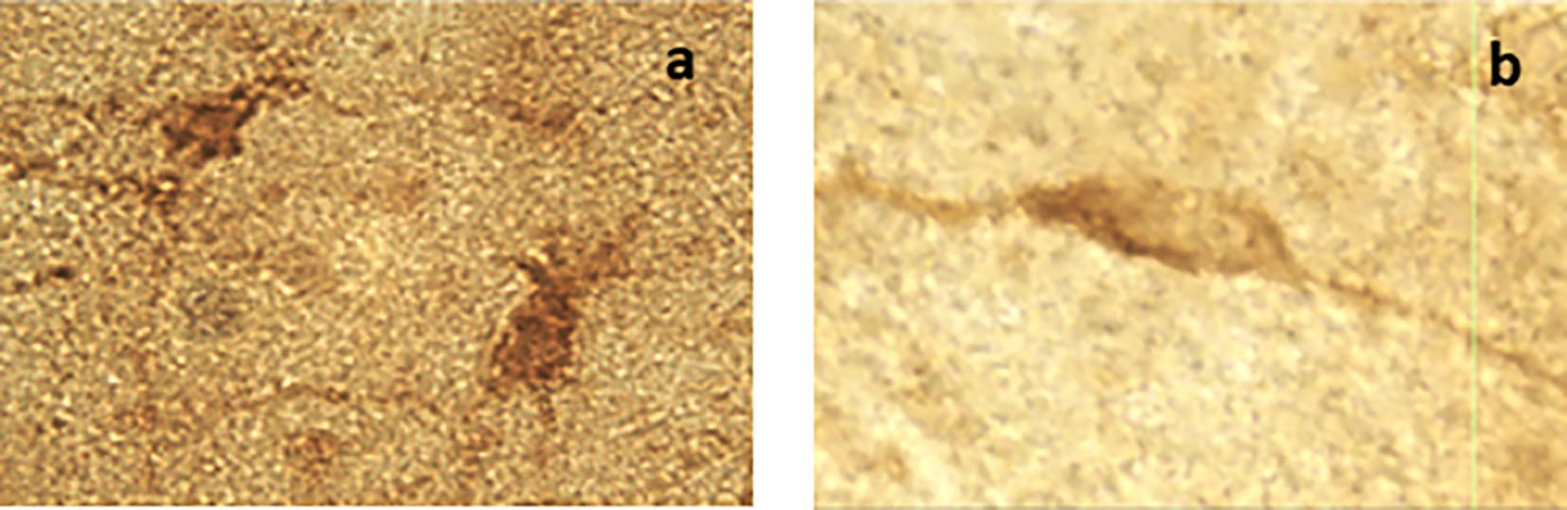
Example photomicrographs of DCX^+^ cells with (**a**) multipolar and (**b**) bipolar morphologies. Images (not to scale) were captured at 100X magnification.

As previously^41,42^, stained DCX^+^ cells were quantified in the rostral (interaural 5.68/0.50) and caudal (interaural 0.50/− 0.50)^72^ HF. An optical microscope (Leica DM6B-Z, Germany) equipped with a digital video camera (Leica DFC450 C, Germany) and motorized stage system (Leica AHM, Germany) was connected to a computer running Stereo Investigator software (version 2018.1.1, MBF Bioscience, USA). HF borders were outlined at 2.5X magnification (0.07 numerical aperture) according to the chick stereotaxic atlas^72^, and cell counting performed at 100X magnification (0.65 numerical aperture) according to the Optical Fractionator method. Stereological parameters were set to an optical fractionator grid of 120 × 120 µm for rostral HF and 240 × 240 µm for caudal HF, a counting frame of 50 × 50 µm for both regions and a mounted thickness of 20 µm. For each animal, 4 to 6 hippocampal Sects. 800 μm apart were systematically analysed, starting with the rostral-most section bearing hippocampal tissue. Quantification was performed blind to housing and body condition groups. DCX^+^ cells of multipolar and bipolar (or fusiform) morphologies were counted separately (see Armstrong et al.^57^). The bipolar/fusiform neurons (medium-small sized cells, elliptical/oval cell bodies, ≤ 2 processes) are assumed to be younger and still migrating, while the multipolar neurons (medium-large sized cells, round or polygonal/angular cell bodies, ≥ 3 processes) are assumed to be more mature and settling^73^. Photomicrographs of example cells with both morphologies are displayed in Fig. 2. Densities of DCX^+^ cells per cubic millimetre of sampled tissue were calculated by dividing the number of counted cells of each type by the area of the counting frame (2500 µm^2^), multiplied by both the number of counting sites sampled in that brain and the section thickness (50 µm), and then multiplying the resulting density per µm^3^ by 10^9^ to obtain a density per mm^3^. Densities for rostral and caudal HF were calculated separately.

### Inflammatory gene expression

The methods used to extract RNA from the sampled spleen tissue, reverse-transcribe it into cDNA, and produce gene-specific templates for standard curves are detailed in Armstrong et al.^74^. Primer sequences for the quantified target genes involved in the inflammatory response (*IL1β, IL6, IL8, IL10* & *TGFβ*) are displayed in Table 1. Quantitative PCR (qPCR) assays were conducted as previously^74^, with *LBR* utilised as a control gene for normalisation. The 48 samples from the adult hens were processed in a single batch. Analyses were performed blind to the group status of the samples. Splenic gene expression was not explored in the sampled pullets. Gene expression values were log(10)- transformed for statistical analysis.

**Table 1:** Primer sequences employed for quantification of inflammatory cytokine expression in splenic tissue.

Gene	Accession	Orientation	Primer Sequence (5′–3′)	Product length (base pairs)
*LBR*	NM_205342	Forward	GGTGTGGGTTCCATTTGTCTACA	80
		Reverse	CTGCAACCGGCCAAGAAA	
*IL1β*	NM_204524.1	Forward	TGCCTGCAGAAGAAGCCTCG	137
		Reverse	CTCCGCAGCAGTTTGGTCAT	
*IL6*	NM_204628.1	Forward	TCGCCTTTCAGACCTACCTG	179
		Reverse	CAGATTGGCGAGGAGGGATT	
*IL8*	NM_204608.1	Forward	TGTGAAGAGATCGCTGTGTG	85
		Reverse	AGGCATCGCATTCCAGC	
*IL10*	NM_001004414.2	Forward	GGGAGCTGAGGGTGAAGTTT	154
		Reverse	TCTGTGTAGAAGCGCAGCAT	
*TGFβ*	NM_001318 456.1	Forward	TTACTACGTGGGCCGGAATG	193
		Reverse	CCCCCAAAAAGGGAACCATCT	

### Composition of the caecal microbiome

For each of the 48 adult hens, DNA was extracted from a 200 mg section of intestinal tissue and caecal content using ZymoBIOMICS DNA minikits (Cambridge Bioscience, UK) according to the manufacturer’s instructions. The section was cut using a sterile scalpel blade to expose the mucosa and luminal contents to bead-beating with a Qiagen TissueLyser at 30 Hz for 10 min. At each extraction, two controls were included, a blank extraction kit to control for contamination and 75 μl of ZymoBIOMICS standard bacterial community (Cambridge Bioscience, UK) to control for variations in DNA extraction efficacy. Extracted DNA was quantified using a NanoDrop 2000 spectrophotometer (NanoDrop Technologies).

Extracted DNA was sent for paired-end sequencing of the 16S rRNA gene at the Centre for Genomic Research (University of Liverpool) using an Illumina MiSeq run. The V4 hypervariable region (515F/R806) was amplified to yield an amplicon of 254 base pairs^75^. Library preparation was performed using a universal tailed tag design with subsequent amplification performed using a two-step PCR with a HiFi Hot Start polymerase (Kapa)^76^. The first round of PCR was performed using the primers 5′ ACACTCTTTCCCTACACGACGCTCTTCCGATCTNNNNNGTGCCAGCMGCCGCGGTAA-3′ (forward) and 5′- GTGACTGGAGTTCAGACGTGTGCTCTTCCGATCTGGACTACHVGGGTWTCTAAT-3′ (reverse)^76^. The raw Fastq files were trimmed for the presence of Illumina adapter sequences using Cutadapt version 1.2.1. The reads were further trimmed using Sickle version 1.200 with a minimum window quality score of 20. Reads shorter than 10 base pairs after trimming were removed.

QIIME2 version 2020.2.0 was used for analysis of the Illumina data^77^. Amplicon sequence variant (ASV) assignment was completed using the dada2 plugin^78^ and a feature table produced using the feature-table plugin^79^. Taxonomy was assigned using a pre-trained NaiveBayes classifier based on the SILVA 132 database of the 515F/R806 region of the 16S rRNA gene^80^, available for download at https://docs.qiime2.org/2018.11/data-resources/, using the q2-feature-classifier plugin^81^.

### Data analysis

Analysis of body and spleen mass, adult hippocampal plasticity and splenic mRNA expression was performed in IBM SPSS Statistics (v.25). Data was confirmed to meet the assumptions of the statistical approaches employed (e.g. by assessing normality of residuals). In the sample of pullets, body masses of the two strains were compared through an independent samples t-test. For spleen mass, univariate ANOVAs were conducted with body mass as a covariate and strain as a between-subject fixed factor. In the adult hens, a univariate ANOVA was used to compare body mass, with housing system and body condition as fixed factors and together in an interaction term. Spleen mass was explored in a similar model, but which also included body mass as a covariate. To explore adult hippocampal plasticity in the pullets, separate linear mixed models (LMMs) with unstructured covariance were conducted for raw DCX^+^ multipolar and bipolar cell densities. These included staining batch as a random factor, HF subregion (rostral vs. caudal) as a repeated fixed factor, and strain (H&N/Hy-Line) as between-subject fixed factor. The interaction between subregion and strain was also included. A univariate ANOVA indicated that DCX^+^ cell densities (all types) did not significantly differ between staining batches for the pullet samples, *F*_1,44_ = 2.98, *p* = 0.061 (Table 2). Densities of stained DCX^+^ cells differed between batches in the samples from adult hens, *F*_1,87_ = 3.03, *p* = 0.014. Descriptive statistics reflecting batch variability are displayed in Table 3. Fewer cells were stained in batch 6 than batch 1 (*p* = 0.009). Other pairwise comparisons were non-significant.

**Table 2:** Mean (M) and standard error (SE) for estimated densities of doublecortin (DCX)^+^ cells of all types (combined multipolar and bipolar morphologies) stained over the whole HF of the 14-week-old laying hen pullets in each of the 3 immunohistochemistry processing batches (balanced for strain), reflecting technical variation.

Batch number (*n* = 8 birds each)	Stained DCX^+^ cell density/mm^3^	
	M	SE
1	2018.79	113.38
2	1875.85	145.95
3	1636.27	51.93

**Table 3:** Mean (M) and standard error (SE) for estimated densities of doublecortin (DCX)^+^ cells of all types (combined multipolar and bipolar morphologies) stained over the whole HF of the adult hens in each of the 6 immunohistochemistry processing batches (balanced for housing systems & body conditions), reflecting technical variation.

Batch number (*n* = 8 birds each)	Stained DCX^+^ cell density/mm^3^	
	M	SE
1	3406.74	633.72
2	2422.60	573.63
3	2653.05	534.45
4	1645.67	246.71
5	2390.62	466.11
6	1147.50	189.24

To analyse cell counts for the adult hens, separate LMMs were again conducted with DCX^+^ multipolar and bipolar cell densities as the dependent variables. Models included staining batch as a random factor, HF subregion as a repeated fixed factor and housing system/strain (multi-tier free range/H&N vs. enriched cage/Hy-Line) and body condition (good vs. poor) as between-subject fixed factors. All interactions between HF subregion, housing system and body condition were included. For the purpose of figures, cell densities were normalised (z-scored) within their staining batch and, due to the consistently higher cell densities quantified in the caudal HF, also within the rostral or caudal subregion. This means the figures don’t allow for a comparison across subregions, but the statistical analysis does.

For each inflammatory gene quantified in the spleen, measured molar mRNA was converted to a ratio of *LBR* mRNA expression in the same sample and log-transformed. A series of univariate ANOVAs were conducted with target transcript expression ratios (*IL10, IL1B, IL6, IL8* or *TGFB / LBR)* as dependent variables, with housing system/strain and body condition as between-subject fixed factors and in an interaction term.

Alpha and beta diversity analyses of extracted caecal DNA were performed at a sampling depth of 13,000 using the alignment^82^, phylogeny^83^ and diversity (https://github.com/qiime2/q2-diversity) plugins. Alpha diversity was measured using Faith’s phylogenetic diversity (FPD) index^84^ to assess species richness and a Shannon diversity (SD) index to assess species evenness. Alpha diversity was compared between sample groups using a Kruskal Wallis test with a false discovery rate (FDR) correction. For numerical data, a Pearson rank correlation was used to assess relationships of other key outcome variables (DCX^+^ cell densities & splenic *IL-6* expression) and alpha diversity. Taxa plots were produced using the q2-taxa plugin (https://github.com/qiime2/q2-taxa). Beta diversity, a metric used to compare species diversity and abundance between samples, was calculated with a robust Aitchison PCA metric^85^ using the DEICODE plugin (https://library.qiime2.org/plugins/deicode). The beta diversity matrix was used to draw principal coordinate analysis (PCoA) plots and an ANOSIM test over 999 permutations were used to determine the significance of differences in beta diversity between groups. Songbird was chosen to analyse differential abundance of ASVs between groups (–*p*-formula “Body_Condition + Housing_System”) since it overcomes challenges created by the compositional nature of microbiota data^86^. Results from both DEICODE and Songbird were visualised through Qurro to identify the taxonomy of ASVs (features) contributing to differences between housing system/strain and body condition groups^87^.

## Results

### Body and spleen mass

Body masses of the 24 sampled pullets in the strain-baseline group were consistent with flock averages at the time of sampling. Mean spleen masses are also displayed in Table 4.

**Table 4:** Descriptive statistics for body and spleen masses of Hy-Line and H&N strain pullets, sampled from the rearing farm to provide a strain-baseline for comparisons between the adult housing systems.

	Hy-Line (n = 12)		H&N (n = 12)	
	M	SE	M	SE
Body mass (kg)	1.28	0.02	1.23	0.02
Spleen mass (g)	2.74	0.17	2.94	0.34

M, mean; SE, standard error.

There was a trend towards the sampled Hy-Line pullets being heavier than their H&N counterparts (*t*_22_ = 1.77, *p* = 0.090). Body mass did not predict spleen mass on an individual basis (*F*_1,21_ = 0.681, *p* = 0.419), and there was no difference in spleen mass between the two strains (*F*_1,21_ = 0.624, *p* = 0.438). One H&N bird had a particularly large spleen, weighing 6.46 g. However, after removal of this outlier, neither body mass nor strain came to predict spleen mass (body mass: *F*_1,20_ = 0.053, *p* = 0.821; strain: *F*_1,20_ = 0.338, *p* = 0.568).

**Figure 3:**
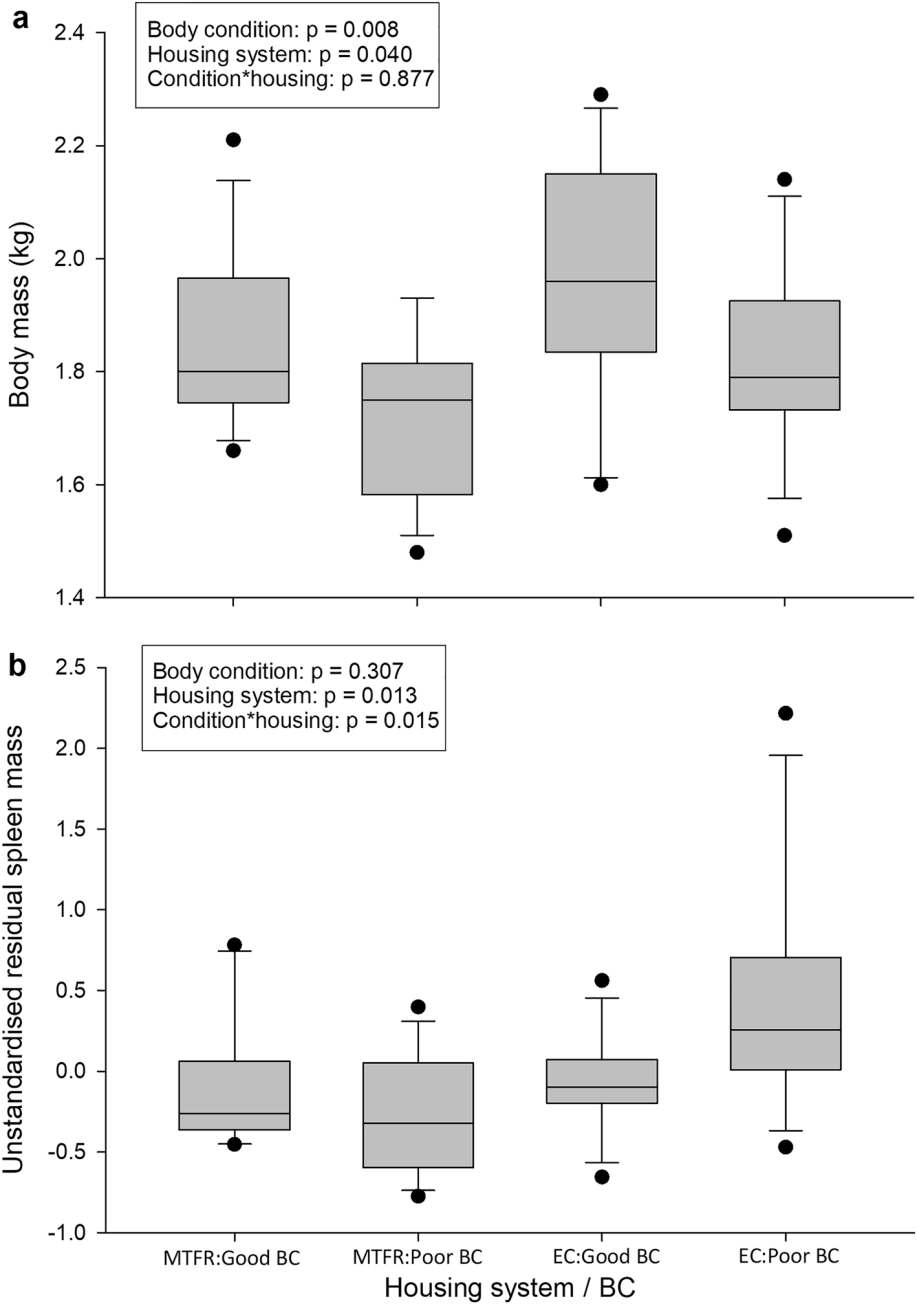
(**a**) body and (**b**) spleen masses of adult hens with good and poor body conditions from a multi-tier free-range (MTFR) system (strain = H&N) and an enriched cage (EC) system (strain = Hy-Line). Boxplots display median, inter-quartile range, 95% confidence intervals and data range. Spleen masses are residual, after controlling for body mass in a simple linear regression. n = 12 for each sub-group.

For the sampled adult hens, H&N strain birds were taken from the multi-tier free-range system and Hy-Line birds were taken from the enriched cage system, Body and spleen masses of good and poor body condition hens are displayed in Fig. 3. As intended, there was a main effect of body condition on body mass, with good condition hens being heavier than their poor condition counterparts (*F*_1,44_ = 7.67, *p* = 0.008). The effect of housing system/strain on body mass was also significant, with birds from the multi-tier free-range (H&N) having lower mass than birds from the enriched cages (Hy-Line; *F*_1,44_ = 4.50, *p* = 0.040). According to breed standards which describe typical performance, Hy-line birds are expected to be heavier than H&N birds^88,89^. There was no interaction between body condition and housing system/strain (*F*_1,44_ = 0.024, *p* = 0.877). In the adult hens, body mass significantly predicted spleen mass as a covariate (*F*_1,43_ = 5.81, *p* = 0.020). There was no main effect of body condition on spleen mass (*F*_1,43_ = 1.07, *p* = 0.307), but a significant effect of housing system/strain, whereby caged birds (Hy-Line) had heavier spleens (*F*_1,43_ = 6.74, *p* = 0.013). This could be ascribed to the poor body condition birds alone (body condition*housing/strain: *F*_1,43_ = 6.41, *p* = 0.015). Specifically, within the enriched cage system (Hy-Line), poor condition birds had heavier spleens than good condition birds (*p* = 0.018), whilst the former group also had higher spleen mass than hens of poor condition in the multi-tier system (H&N; *p* = 0.001). In contrast, no difference in spleen mass was present between housing systems/strains for birds in good condition (*p* = 0.895).

### Adult hippocampal plasticity

**Figure 4:**
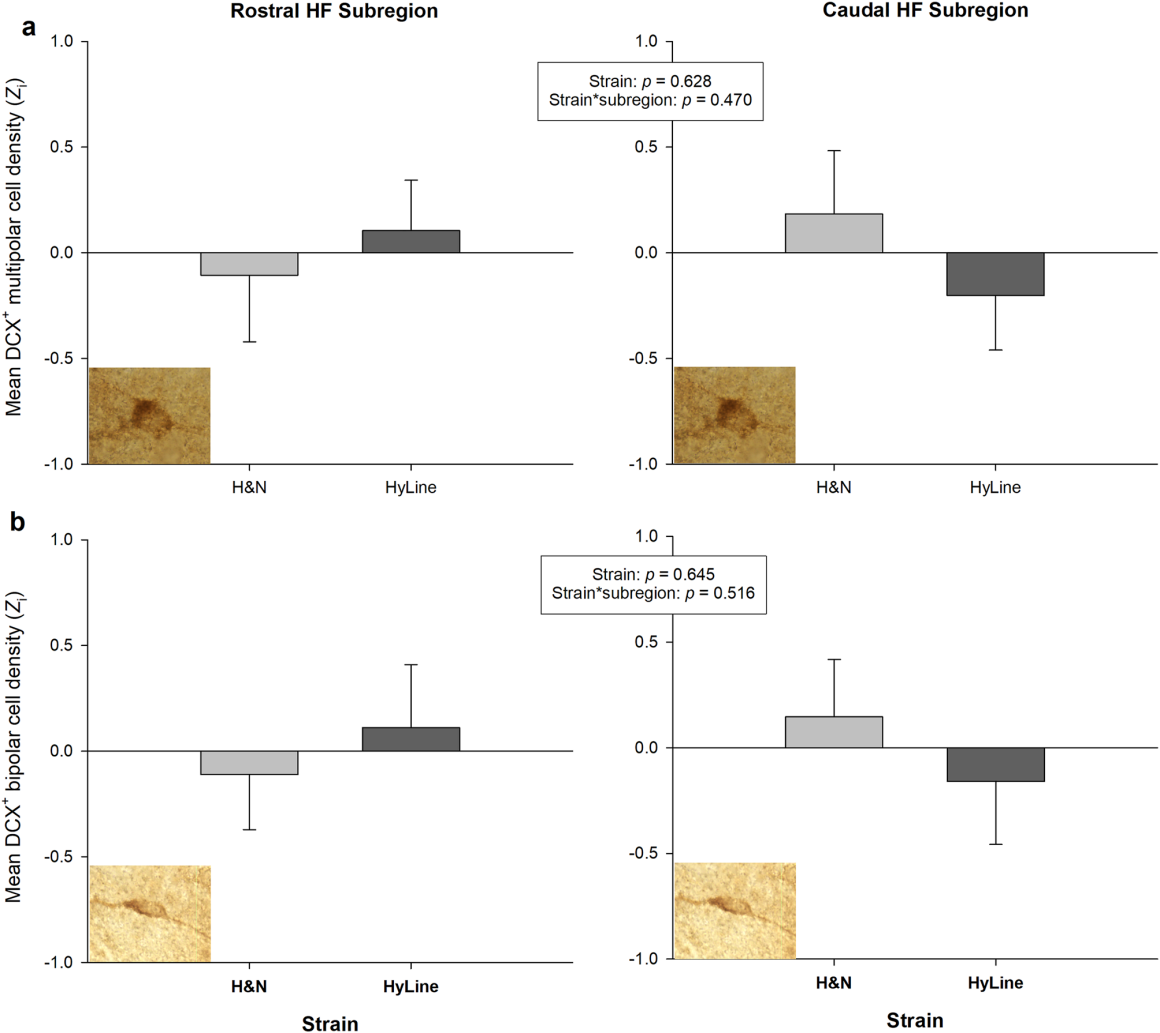
Mean densities of cells expressing doublecortin (DCX) with (**a**) multipolar, and (**b**) bipolar (or fusiform) morphology, in the rostral and caudal hippocampal formation (HF) subregions of H&N (n = 12) and Hy-Line (n = 12) strain laying hen pullets (14 weeks old). Error bars display + 1 standard error. Cell densities are normalized within staining batches and subregion of the HF (rostral/caudal) using the standard score (*Z*_i_). The figure therefore does not allow a direct comparison between the two HF subregions, only between strains within subregion. Raw densities were higher in the caudal HF than the rostral HF for DCX^+^ multipolar (*p* < 0.001) and bipolar (*p* = 0.021) cells (see “Adult Hippocampal Plasticity” section). Cell densities did not differ between the strains, and there was no interaction with HF subregion.

Descriptive statistics for subregional DCX^+^ cell densities in the two sampled strains of 14-week-old laying hen pullet are displayed in Table 5. A greater density of DCX^+^ multipolar (*F*_1,22.1_ = 29.2, *p* < 0.001) and bipolar (*F*_1,21.3_ = 6.21, *p* = 0.021) cells were quantified in the caudal HF than in the rostral subregion. The H&N and Hy-Line pullet strains did not differ from each other in their densities of DCX^+^ multipolar cells over the whole HF (*F*_1,19.4_ = 0.242, *p* = 0.628, Fig. 4a), and there was no interaction between strain and HF subregion (*F*_1,22.1_ = 0.541, *p* = 0.470). Densities of DCX^+^ bipolar cells also did not differ between the two strains (*F*_1,21.0_ = 0.219, *p* = 0.645, Fig. 4b), and strain did not interact with HF subregion to predict bipolar densities (*F*_1,21.3_ = 0.437, *p* = 0.516).

**Table 5:** Mean (M) and standard error (SE) for estimated densities of doublecortin (DCX)^+^ cells with multipolar and bipolar morphologies in the rostral and caudal hippocampal formation (HF) of 14-week-old H&N and Hy-Line strain laying hen pullets.

Strain	Multipolar DCX^+^ cell density (/mm^3^)				Bipolar DCX^+^ cell density (/mm^3^)			
	Rostral HF		Caudal HF		Rostral HF		Caudal HF	
	Mean	SE	Mean	SE	Mean	SE	Mean	SE
H&N	432.66	35.65	680.55	52.54	1158.44	35.53	1149.58	104.12
Hy-Line	446.77	32.01	626.80	52.97	1185.13	47.80	1368.66	116.85

*n* = 12 for each strain.

**Figure 5:**
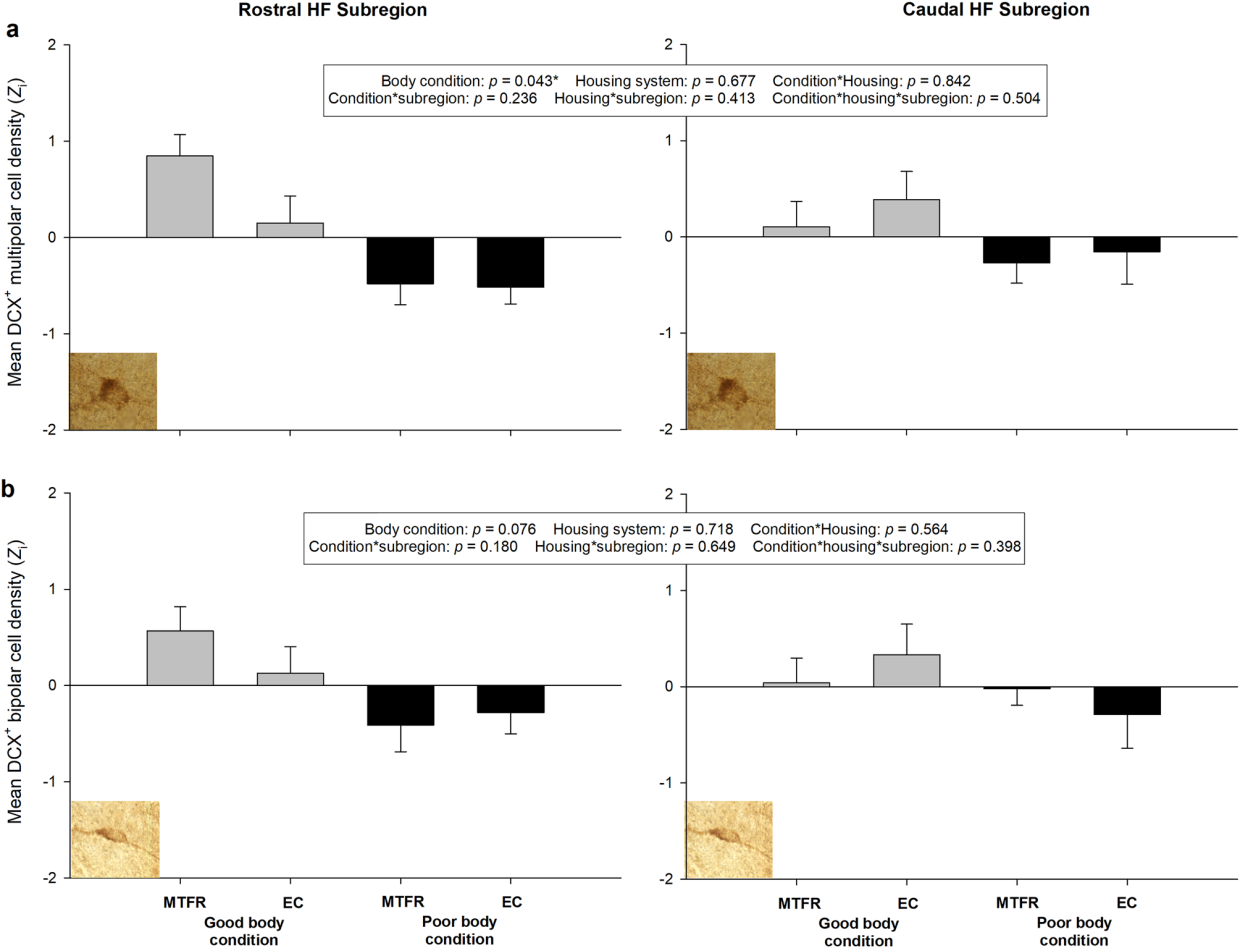
Mean densities of cells expressing doublecortin (DCX) with a) multipolar, and b) bipolar (or fusiform) morphology, in the rostral and caudal hippocampal formation (HF) subregions of adult commercial laying hens, grouped according to housing system (multi-tier free range (MTFR) versus enriched cage (EC)) and physical body condition (good vs. poor BC). n = 12 for each sub-group. Error bars display + 1 standard error. Cell densities are normalized within staining batches and subregion of the HF (rostral/caudal) using the standard score (*Z*_i_). The figure therefore does not allow a direct comparison between the two HF subregions, only between body conditions and housing systems within subregion. Raw densities were higher in the caudal HF than the rostral HF for DCX^+^ multipolar and bipolar cells (*p* < 0.001, see “Adult Hippocampal Plasticity” section). Poor BC hens had lower multipolar cell densities over the whole HF than hens with good body condition*.

Descriptive statistics for DCX^+^ cell densities in the sampled groups of adult birds are displayed in Table 6. A higher density of DCX^+^ multipolar neurons was again found in the caudal HF than in the rostral subregion (*F*_1,41.5_ = 57.07, *p* < 0.001). There was no main effect of housing system/strain (multi-tier system = H&N, enriched cage = Hy-Line) on multipolar cell density (*F*_1,38.9_ = 0.176, p = 0.677), but hens with poor body condition had lower multipolar cell densities over the whole HF than hens with good body condition (*F*_1,39.0_ = 4.36, *p* = 0.043, Fig. 5a). There was no interaction between housing system/strain and body condition (*F*_1,38.9_ = 0.040, *p* = 0.842), nor did HF subregion interact with housing system/strain (*F*_1,41.6_ = 0.684, *p* = 0.413) or body condition (*F*_1,41.5_ = 1.45, *p* = 0.236). Finally, there was no three-way interaction between HF subregion, housing system/strain and body condition for multipolar cell densities (*F*_1,41.6_ = 0.454, *p* = 0.504). The density of DCX^+^ bipolar cells was also higher at the caudal HF pole than in the rostral region (*F*_1,41.6_ = 53.00, *p* < 0.001). Housing system/strain also had no main effect on bipolar cell densities (*F*_1,38.6_ = 0.132, *p* = 0.718). There was a trend towards hens with poor body condition having a lower density of DCX^+^ bipolar cells than their good condition counterparts (*F*_1,38.7_ = 3.32, *p* = 0.076, Fig. 5b). There was no interaction between housing system/strain and body condition (*F*_1,38.6_ = 0.338, *p* = 0.564), and HF subregion did not interact with housing system/strain (*F*_1,41.7_ = 0.211, *p* = 0.649) or body condition (*F*_1,41.6_ = 1.86, *p* = 0.180). Also, there was no three-way interaction between HF subregion, housing system/strain and body condition on bipolar cell densities (*F*_1,41.7_ = 0.730, *p* = 0.398).

**Table 6:** Mean (M) and standard error (SE) for estimated densities of doublecortin (DCX)^+^ cells with multipolar and bipolar morphologies in the rostral and caudal hippocampal formation (HF) of adult laying hens, grouped according to housing system (multi-tier free range (MTFR) versus enriched cage (EC)) and physical body condition (good/poor BC).

		Multipolar DCX^+^ cell density (cells/mm^3^)				Bipolar DCX^+^ cell density (cells/mm^3^)			
		Rostral HF		Caudal HF		Rostral HF		Caudal HF	
Housing (*strain*)	Body condition	M	SE	M	SE	M	SE	M	SE
MTFR (*H&N*)	Good	504.67	86.73	1462.07	247.50	837.40	59.09	2181.77	330.25
	Poor	333.13	74.21	1155.40	166.42	711.70	118.87	1885.96	241.4
EC (*HyLine*)	Good	384.93	39.02	1751.58	386.77	761.03	53.24	2615.77	500.49
	Poor	340.84	68.07	1203.75	298.20	746.52	93.32	1773.61	425.11

*n* = 12 for each sub-group.

### Expression of mRNA for inflammatory cytokines in the spleen

**Figure 6:**
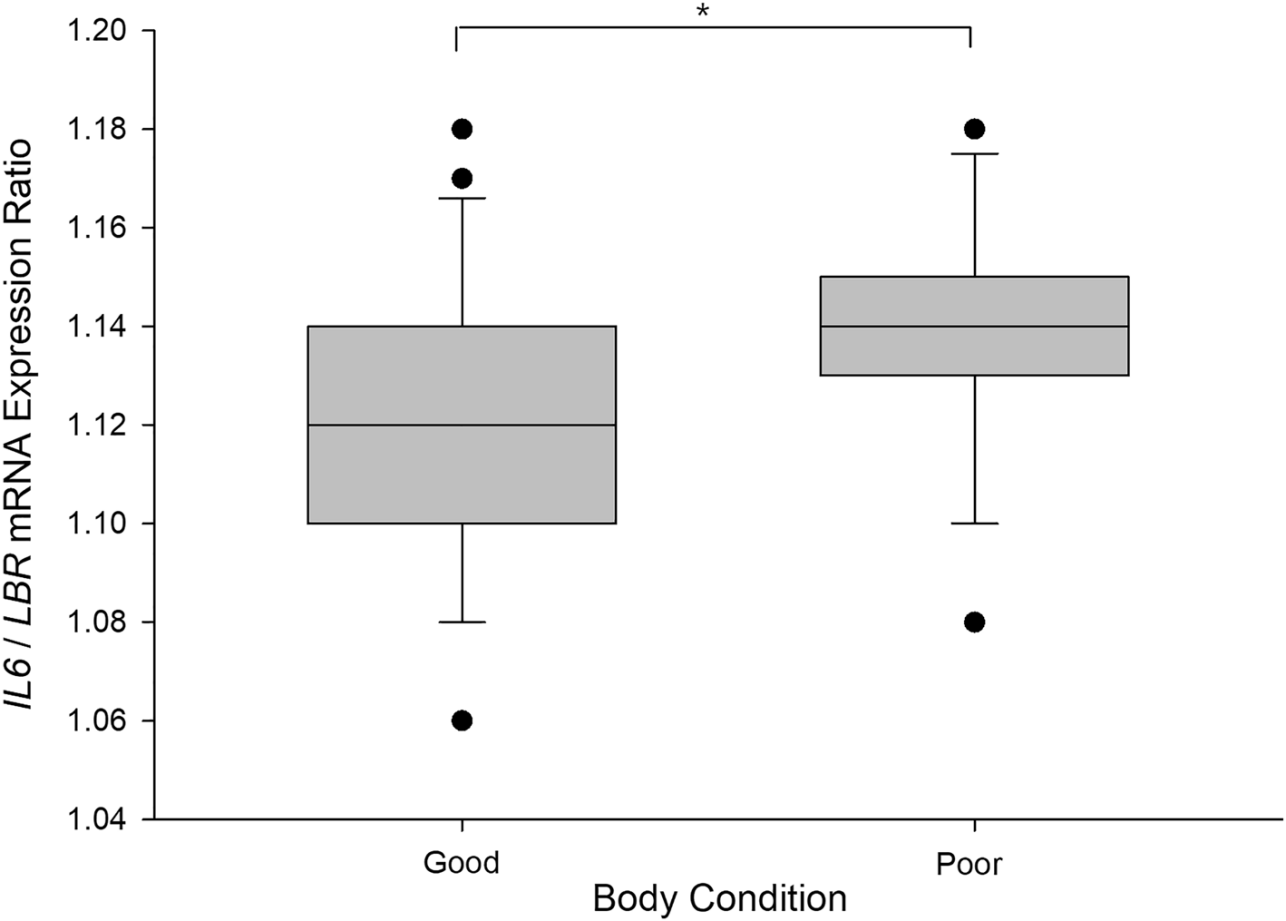
Ratio of expression of mRNA for interleukin-6 (*IL-6*) relative to housekeeping gene *LBR* in the spleens of adult laying hens with good (n = 24) versus poor (n = 24) physical body condition (BC). Boxplots display median, inter-quartile range, 95% confidence intervals and data range. Splenic *IL-6* mRNA expression was higher in poor BC hens than in good BC hens, *p* = 0.026*.

Expression of control gene *LBR* in the spleens of the adult hens did not differ with housing system/strain (*F*_1,44_ = 0.81, *p* = 0.372) or body condition (*F*_1,44_ = 0.006, *p* = 0.936), nor did these factors interact (*F*_1,44_ = 0.22, *p* = 0.641). Inflammatory gene expression also did not differ between the two housing systems/strains (multi-tier = H&N, enriched cage = Hy-Line) for *IL-10* (*F*_1,42_ = 0.07, *p* = 0.800), *IL-1β* (*F*_1,42_ = 0.18, *p* = 0.670), *IL-6* (*F*_1,43_ = 1.50, *p* = 0.228), *IL-8* (*F*_1,41_ = 0.04, *p* = 0.852), or *TGFβ* (*F*_1,42_ = 0.10, *p* = 0.756). There was no effect of body condition on expression of *IL-10* (*F*_1,42_ = 0.18, *p* = 0.676), *IL1β* (*F*_1,42_ < 0.001, *p* = 0.991), *IL-8* (*F*_1,41_ = 1.06, *p* = 0.308), or *TGFβ* (*F*_1,42_ = 0.004, *p* = 0.950). However, splenic expression of *IL-6* mRNA was higher in hens with poor body condition (M = 1.137, SEM = 0.005) than in hens with good body condition (M = 1.119, SEM = 0.006, *F*_1,43_ = 5.29, *p* = 0.026, Fig. 6). There were no significant interactions between housing/strain and body condition (*p* ≥ 0.491).

When included as a covariate in the previous model, splenic *IL-6* expression ratio did not correlate with DCX^+^ multipolar cell densities in the HF of individual hens (*F*_1,37.7_ = 0.36, *p* = 0.550). The same was true for bipolar cells (*F*_1,38.2_ = 0.90, *p* = 0.349).

### Composition of the caecal microbiome

#### Sequencing effort

Sufficient DNA for 16S rRNA sequencing was extracted from all samples. A total of 7,443,396 reads were obtained. After filtering, merging of paired reads and chimera removal, a total of 4,988,403 reads remained (67% of the original total) giving a mean of 69,283 (± 13,979) reads per sample. The median number of reads per sample was 69,415.

#### Effect of housing system and body condition on alpha and beta diversity

Housing system/strain (multi-tier = H&N, enriched cage = Hy-Line) had a significant impact on mean Faith’s Phylogenetic Diversity (FPD; test statistic = 13.8, *p* = 0.0002) and Shannon Diversity (SD; test statistic = 3.76, *p* = 0.05) with higher FPD and SD in chickens from multi-tier housing (H&N). There was no significant difference in mean FPD index (test statistic = 1.53, *p* = 0.22) or SD (test statistic = 0.58, *p* = 0.45) between good and poor body condition chickens. Other variables included in the analysis were not correlated with alpha diversity: DCX^+^ cell counts in the caudal (*r* = -0.04, *p* = 0.79) and rostral (*r* = 0.11, *p* = 0.48) HF; splenic *IL-6* mRNA expression (*r* = 0.20 *p* = 0.19).

**Figure 7:**
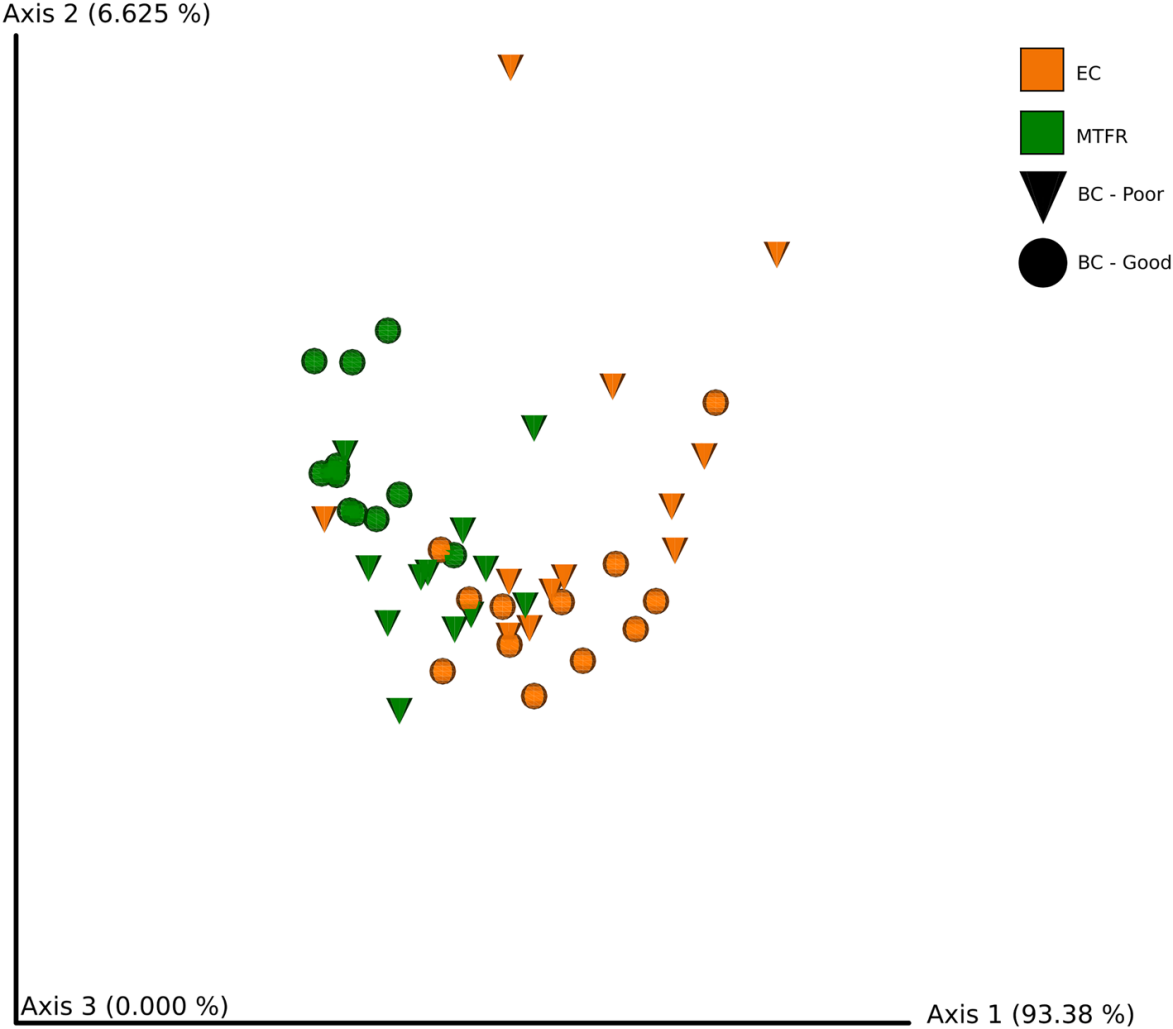
PCoA plot showing robust Aitchison distance between samples from enriched cage (EC, orange) and multi-tier free range (MTFR, green) chickens with poor (triangle) and good (circle) body condition. There is clustering of samples by housing system, with some subdivision by body condition score.

Housing system/strain had a significant effect on beta diversity (test statistic = 45.3, *p* = 0.001), but body condition did not (test statistic = 2.26, *p* = 0.15). A PCoA plot of robust Aitchison distance between samples showed some clustering of samples by housing system/strain and body condition (Fig. 7). There was separation of chickens from the multi-tier system (H&N) with good body condition and other categories, with chickens from the multi-tier system with poor body condition being more similar to all chickens from the enriched cages (Hy-Line). Given that there appeared to be separate clustering of chickens from different body condition groups in the multi-tier housing, robust Aitchison distance was calculated for samples from the two systems separately. When samples from different housing systems were considered separately, there was a significant effect of body condition on beta diversity in the multi-tier aviary (H&N strain hens; test statistic = 27.2, *p* = 0.001) but not in the enriched cages (Hy-Line strain hens; test statistic = 0.33, *p* = 0.57). Beta diversity was not significantly predicted by DCX^+^ cell densities in the caudal (test statistic = 4.2, *p* = 0.06) or rostral (test statistic = 0.005, *p* = 0.97) HF, or by *IL-6* expression (test statistic = 3.48, *p* = 0.07).

#### Differential features between housing system and body condition

**Figure 8:**
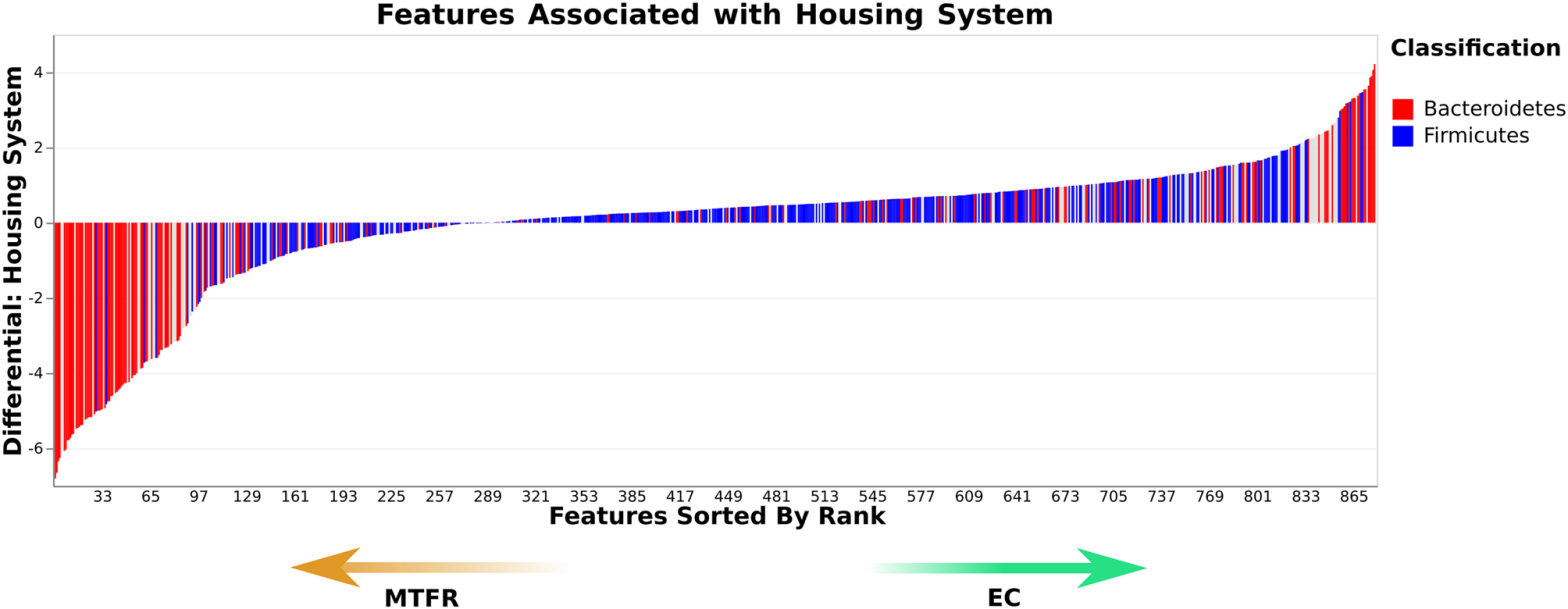
A plot of differential rankings of features that contribute to differences in caecal microbiota composition between MTFR and EC housed chickens. Most features that were highly differentiating for MTFR were assigned to Bacteroidetes with more Firmicutes ranked as differential for EC.

**Figure 9:**
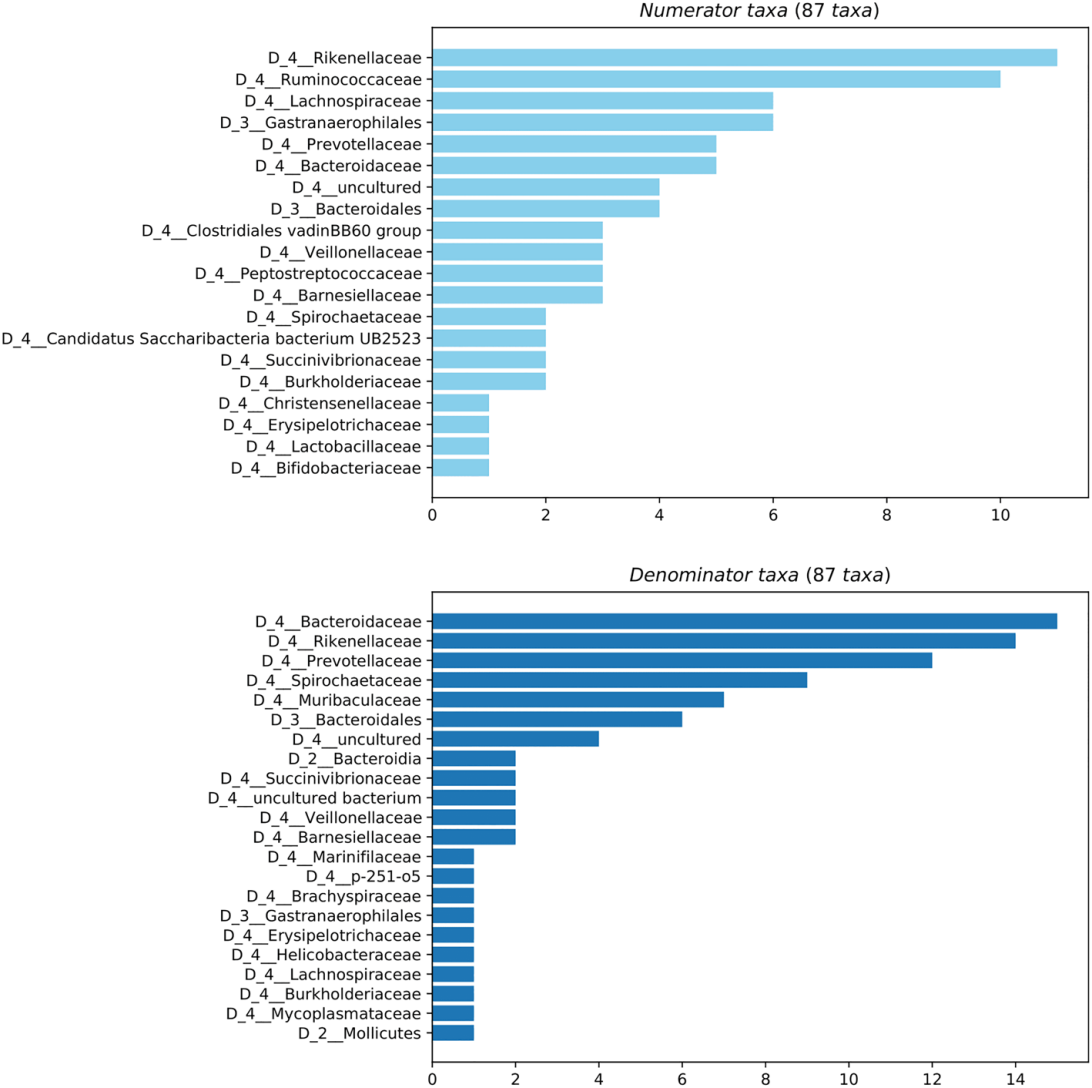
Taxonomy of the top and bottom 10% of discriminating features between housing systems. A log ratio of the relative abundance of these features was significantly higher in EC samples demonstrating an increased proportion of numerator features compared to denominator features.

Songbird analysis included 878 features. First the effect of housing system/strain was considered. A common dichotomy in the chicken caecal microbiome is the balance between *Bacteroidetes* and *Firmicutes*^90^. These taxa were used as the numerator and denominator of log ratios respectively. The log ratio was significantly lower in enriched-cage (Hy-Line) chickens (test statistic = 2.43, *p* = 0.019) indicating a higher proportion of *Firmicutes* compared to *Bacteroidetes* in these animals. From a plot of differential rankings of features (Fig. 8), most features that were highly differentiating for multi-tier (H&N) birds were *Bacteroidetes,* with more *Firmicutes* ranked as differential for enriched cage (Hy-Line) birds. To explore other taxa contributing to differences between housing systems, the top and bottom 10% of discriminating features (n = 87) between housing systems were selected as the numerator and denominator features. The taxonomic family of these features is shown in Fig. 9. The log ratio was significantly higher in enriched-cage (Hy-Line) samples (test statistic = 27.8, *p* < 0.001), indicating a higher proportion of numerator features, with a higher proportion of denominator features in multi-tier (H&N) samples. A greater number of *Cyanobacteria* were present in the numerator features (higher in enriched-cage samples) while a higher number of *Spirochaetes* were present in the denominator features (higher in multi-tier samples). Within the features assigned to *Spirochaetes*, those further identified as *Treponema* were only identified as differential in multi-tier (Hy-Line) samples.

**Figure 10:**
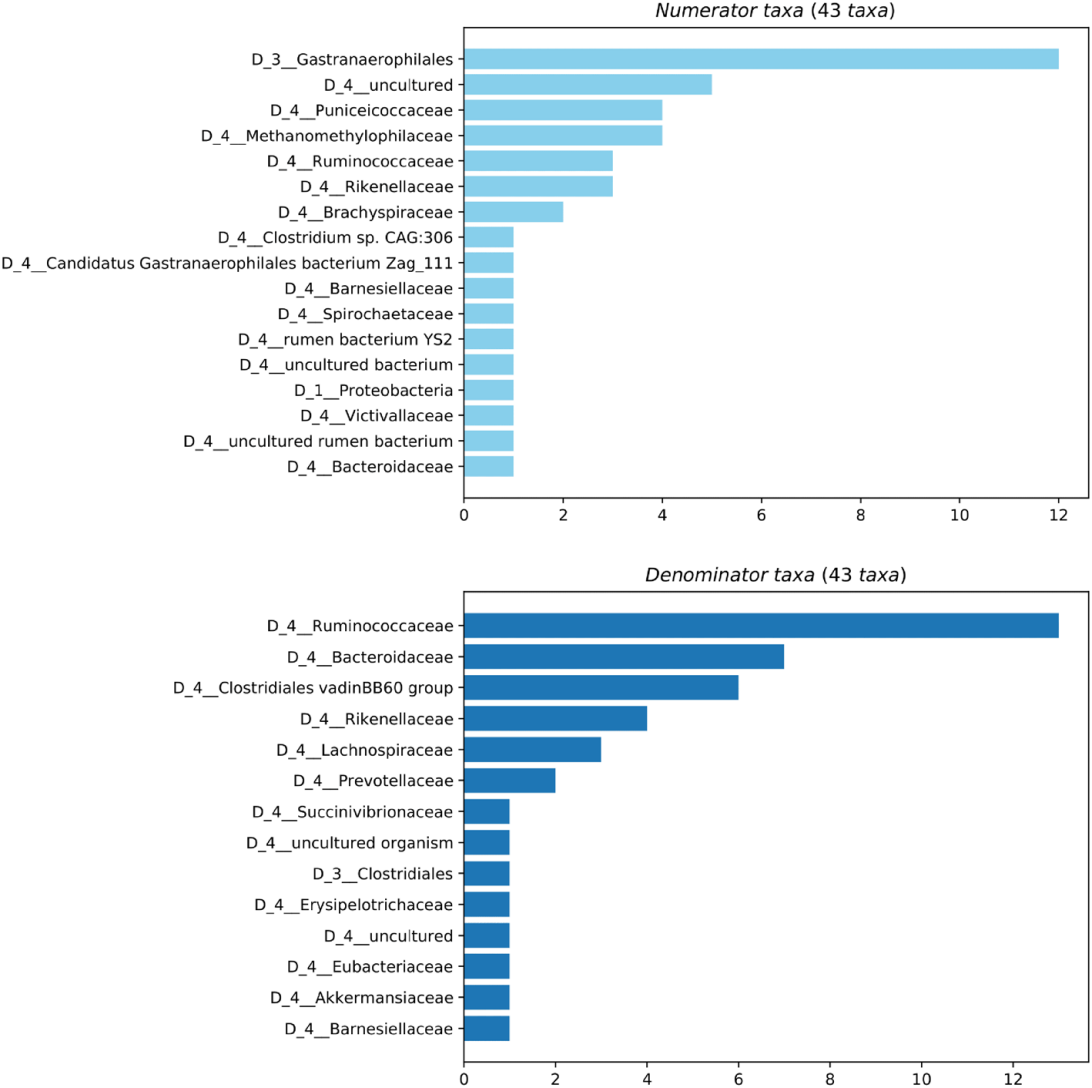
Taxonomy of the top and bottom 5% of discriminating features between samples from good and poor body condition chickens. A log ratio of the relative abundance of these features was significantly higher in poor body condition samples indicating an increased proportion of numerator features.

**Figure 11:**
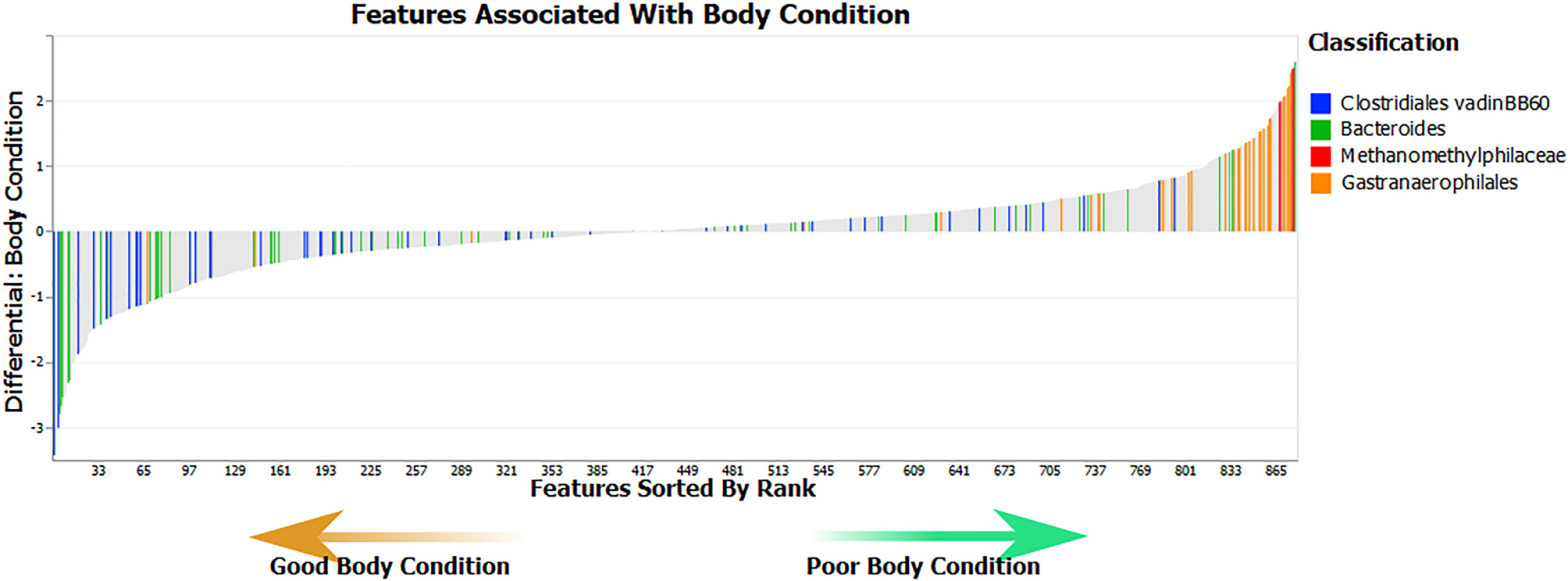
A plot of differential rankings of features that contribute to differences in caecal microbiota composition between chickens with good and poor body condition. Features assigned to Gastranaerophilales and Methanomethylphilaceae were highly differentiating for poor body condition while those assigned to Clostridiales vadinBB60 and Bacteroides were differentiating for good body condition.

Features that were differentially abundant between chickens of different body conditions were explored further. Given that the *Bacteroidetes:Firmicutes* ratio has previously been postulated to affect host metabolism^91^, the log ratio of these taxa was explored for an effect of body condition. There was no significant difference in the log ratio of *Bacteroidetes* and *Firmicutes* between good condition and poor condition hens (test statistic = 0.05, *p* = 0.96). The top and bottom 5% of discriminating features (n = 43) between body condition groups were selected as the numerator and denominator features. The taxonomic family of these features is shown in Fig. 10. The log ratio was significantly higher in poor body condition samples (test statistic = 7.1, *p* < 0.001), indicating an increased proportion of numerator features, with a higher proportion of denominator features in good body condition samples. Features assigned to *Cyanobacteria*, *Euryarchaeota*, *Spirochaetes* and *Lentisphaerae* were associated with poor body condition while features assigned to *Firmicutes* and *Bacteroidetes* were more associated with good condition samples. The taxonomy of features associated with poor body condition were explored further. Those assigned to *Cyanobacteria* could only be assigned to the level of order as *Gastranaerophilales*. The four features assigned to *Euryarchaeota* all belonged to the family *Methanomethylophilaceae* and two of the features assigned to *Spirochaetes* were assigned to the genus *Brachyspira*. Further exploration of the taxonomy of features associated with good body condition showed a high proportion of features assigned to the family *Clostridiaceae vadinBB60* and genus *Bacteroides*. The distribution of these taxa within the feature rankings is shown in Fig. 11.

## Discussion

Within both enriched cage and multi-tier aviary housing, all measures of chronic stress taken differed between adult hens with good and poor physical body conditions. Poor condition hens exhibited lower densities of multipolar neurons expressing DCX, elevated splenic expression of inflammatory cytokine *IL-6*, and a higher abundance of caecal microbiota such as *Methanomethylophilaceae*. Microbial differences were also observed between hens from the two housing systems, potentially relating to exposure to a greater diversity of microbes occurring within the multi-tier free-range system, but these groups did not differ in their average DCX^+^ cell densities or inflammatory gene expression. The results suggest that, within laying hen flocks, poor condition hens experience relatively more chronic stress than their good condition counterparts. In contrast, stress profiles appear comparable between housing systems for hens at both the top and bottom of the body condition spectrum, suggesting that the range of stressful experience may be similar for flocks housed in enriched cages and multi-tier free-range systems. Because the two sampled housing systems contained adult hens of different strains (H&N and Hy-Line), a potential strain difference in adult hippocampal plasticity was assessed in a separate group of 14-week-old pullets from the rearing farm. As baseline DCX^+^ cell densities were comparable, it is unlikely that a genetic difference in initial hippocampal plasticity obscured a subsequent disparity in adult stress.

Within housing and strains, results of the body condition comparison indicate either that prolonged stress results in a phenotype characterised by low body mass, sparse feathering, and a pale comb, and/or that being in a poor physical state is chronically stressful for commercial hens. The correlational nature of the study precludes a definitive conclusion about the causal direction of this association, though it is likely that cumulative chronic stress has a detrimental impact on both body condition and adult hippocampal plasticity. Immune responses (e.g. to infectious agents) activate the hypothalamic–pituitary–adrenal (HPA) stress axis^92^, and a contribution of ill health to the observed suppression in adult hippocampal plasticity is supported by the other measures taken. In the enriched cages, poor condition hens had enlarged spleens, consistent with the presence of acute infection^93,94^. Poor condition hens from both types of housing also presented elevated expression of *IL-6* mRNA. IL-6 is one of the primary proinflammatory cytokines driving activation of the HPA-axis in response to immune challenge^95^, and chronically elevated expression of such cytokines is associated with inflammation and poor welfare^59^. In mice, IL-6 expression is also upregulated by repeated social stress, in a response blocked by anxiolytic treatment^96^. In chickens, IL-6 expression is known to be increased by heat^97^ and cold^98^ stress, Cort administration^61^ and infection^99^. While this finding supports the contribution of illness to poor body condition, social stressors such as the receipt of aggressive pecks, reflected by poor feathering^37^, might also add to inflammation.

Generally, gut microbiota interact in complex, bidirectional ways with the nervous and immune systems^100^. Microbiome composition is altered by stress^101^ and has reciprocal influences on stress-related behaviour and physiology^102–104^. Significant differences in caecal microbiota were present between hens with good and poor body conditions. Most amplicon sequence variants that were more abundant in poor condition birds were assigned to *Gastranaerophilales*, a poorly characterised taxon that makes interpretation of the result difficult. However, in both types of housing, poor body condition hens also had a higher abundance of methanogenic archea *Methanomethylophilaceae*. In ruminants, production of methane represents an energy loss of up to 12% of gross energy intake^105^, which in theory could be used for growth and production. Given that the bovine rumen and chicken caecum are functionally analogous bacterial fermentation chambers for plant material, the presence of methanogenic archea may also represent a net energy loss to the chicken. If this is the case, as further studies of chicken host metabolism may determine, then higher levels of methanogens likely contribute to poorer body condition. Furthermore, the identification of *Brachyspira* in the microbiome of some poor body condition birds may also represent a direct cause of this state, as a number of species of this genus lead to intestinal spirochaetosis, with associated weight loss, splenomegaly and loss of body condition in laying hens^106^.

There was no main effect of housing system on adult hippocampal plasticity or inflammatory gene expression. This result is consistent with previous conclusions that no single type of commercial housing system is ideal from the perspective of hen welfare, as each is associated with unique limitations^1,2^. Notably, Rodenburg et al.^1^ compared flocks from several enriched cage and non-cage systems and found no difference in an integrated welfare score that collated multiple indicators, including fearfulness, plumage, body and bone condition, dust levels and mortality. As with the sampled flocks in the present study, group size usually differs dramatically between enriched cage and aviary systems, but it is not clear which social system hens would prefer^107,108^. Multi-tier (also known as aviary) systems are more complex and facilitate greater activity^1,2^, but cannibalism and other deleterious behaviours (e.g. piling and smothering) may be more prevalent and difficult to control than in enriched cages^2,4,109^. Non-cage systems are generally associated with an increased incidence of keel bone fractures^1,2^, but birds exhibiting such damage were excluded from the present sample due to thorough exploration of this welfare issue in our previous study^57^. Though not true for the flocks sampled here, greater mortality has been observed in litter-based (i.e. non-cage) housing than enriched cages^1,110^ and associated with higher airborne concentrations of dust and bacteria^1^ and greater average prevalence of bacterial and parasitic diseases^4,111^ in the former. Contact with infectious microorganisms in free-range systems is further increased by exposure to soil and transfer from wild animals^2^. Overall, trade-offs between different positive and negative aspects of both systems may lead to little net difference in chronic stressful experience for hens. In the case of hippocampal plasticity levels, cognitive stimulation^112,113^ and stress^56^ have opposing directional influences, so comparable DCX^+^ cell densities may reflect the overall balance between environmental complexity and negative experience in each housing system, rather than relative stress alone.

Observed differences in composition of the microbiome between the sampled housing systems appear to reflect these environments. The broad pattern was for increased *Bacteroidetes* in the multi-tier free-range and increased *Firmicutes* in the enriched cages. Because most *Firmicutes* genomes contain genes enabling sporulation, whereas *Bacteroidetes* are not spore-forming but microaerotolerant (able to survive up to 24 h)^114^, there is a relative time limit on the survival of *Bacteroidetes* within a system. An experiment observing bacterial colonisation of chicks housed with an adult hen further demonstrated that *Bacteroidetes* were passed from the hen to the chicks, but *Firmicutes* such as *Lachnospiraceae* and *Ruminococcaceae* were derived from the environment^115^. It is therefore logical that taxa such as *Bacteroidetes* sustain a greater diversity and abundance within the microbiota in housing systems such as the multi-tier free-range because their design permits contact between larger numbers of individuals. In the present study, differences in caecal microbiota composition cannot be attributed solely to housing system design, as uncontrolled factors such as local exposure to different bacterial profiles^116^ will have doubtless contributed. Though potential effects of strain should also not be discounted, it has been concluded that environmental exposure, diet and management practices are more influential than genetics in shaping the caecal microbiota^116,117^.

A limitation of the present comparison is that the multi-tier housing contained H&N strain hens, while the enriched cage system housed Hy-Line birds. Though no baseline difference in adult hippocampal plasticity was observed between pullets of the two strains, differences in age-dependent adult hippocampal plasticity and/or in lifetime stress resilience cannot be ruled out. The lack of observed difference between multi-tier H&N hens and enriched-cage Hy-Line hens suggests an overall difference in hippocampal neurogenesis may not exist between the two strains later in life, but the design of the study does not permit assessment of possible interactions between strain and other factors (e.g. body condition). Inflammatory gene expression was also not measured in the pullets, though unlike the adult birds, they exhibited no group difference in relative spleen mass. Given that only one flock was sampled from each type of housing system, it is also possible that the flocks sampled were relatively good or poor welfare examples of enriched cage and multi-tier free range systems. Future studies should assess differences between housing systems over a larger number of representative flocks. Additionally, as no experimental manipulations were conducted, the causal relationships cannot be conclusively determined. While it is presumed that changes in adult hippocampal plasticity between the body condition groups are a result of prolonged experience, as in Gualtieri et al.^56^, it is theoretically possible that the influence of existing individual differences in adult hippocampal neurogenesis on personality and behaviour manifested in differences in external physical condition between hens.

A threshold for the requisite level of stress to elicit a measurable change in adult hippocampal plasticity may exist. On a technical level, variability between staining batches in the present study may also have introduced some ceiling effects in terms of the proportion of cells labelled, possibly limiting power to detect a housing system effect. Nevertheless, at the extremes of the body condition distribution, individual variation within systems appears to exceed shared inter-system effects of the housing environment on cumulative chronic stress. This finding has several implications. Interventions targeted at improving flock body condition, particularly for those birds in the poorest physical states, may have the greatest benefit for overall welfare. Controlled experiments to determine which factors are most associated with poor body condition and may therefore contribute most to long-term stress may further inform these measures. Selection for innate immunity and stress resilience may also have advantages for the subjective experience of laying hens, improving their collective chances of having a “life worth living”^118^. Because the distribution of hens with different body conditions may not be comparable across systems, population level stressful experience may still differ between the sampled housing types. Importantly, our results suggest that future assessment of comparative welfare between housing systems, husbandry practices and flocks may be facilitated by systematic sampling to categorise birds according to external body condition. Measuring the body condition distribution associated with particular housing systems could provide a corresponding proxy “stress profile”.

## Conclusions

In an enriched cage and non-cage (multi-tier aviary) commercial housing system, hens in poor physical condition exhibit a lower level of adult hippocampal plasticity than hens in good condition. This suggests that the cumulative chronic stress experienced by these individual hens over the laying period is greater. Poor condition hens also displayed elevated splenic expression of *IL-6*, differing microbial compositions (including an increased abundance of *Methanomethylophilaceae* and presence of *Brachyspira*), and enlarged spleens in the enriched cages. These factors point to associations between physical health, inflammation, chronic stress and body condition. A lack of main effect of housing type on adult hippocampal plasticity suggests that the range of stressful experience for hens in the multi-tier aviary and enriched cage housing is similar, or at least below the threshold for detectable differences, but only one replicate house per system was used here. Assessing whether experience for an average hen in each system differs will require systematic sampling of body condition distribution. Overall, our findings suggest that housing systems (and husbandry practices) which produce the lowest proportion of birds with poor body conditions may be associated with the “best” relative overall welfare. Measures to promote and maintain good body condition will have positive consequences for hen welfare.

## Data Availability

Microbiome sequencing data is available on the NCBI Sequence Repository Archive, under BioProject Number PRJNA762251. The other datasets generated and analysed during the study are stored in a publicly accessible repository, at: https://doi.org/10.25405/data.ncl.14135153.
